# Cyberbullying across the Lifespan of Education: Issues and Interventions from School to University

**DOI:** 10.3390/ijerph16071217

**Published:** 2019-04-04

**Authors:** Carrie-Anne Myers, Helen Cowie

**Affiliations:** 1 Department of Sociology, City, University of London, London EC1V 0HB, UK; 2 Department of Health and Medical Sciences, University of Surrey, Guildford GU2 7XH, UK; H.Cowie@surrey.ac.uk

**Keywords:** cyberbullying, peer support, bystanders, moral disengagement, cyberbullying and the law, mental health, social environment, cyberbullying interventions, educational lifespan

## Abstract

Research on cyberbullying amongst students has tended to be conducted separately within specific education institutional contexts, schools, further education (FE) and higher education (HE), neglecting a view that takes account of the entire educational lifespan. The present article addresses this gap in the literature, providing a novel take on examining its nature, social environments, legal consequences and potentially helpful interventions. To facilitate this, the article conceptualises cyberbullying in broad terms, recognising that it can take multiple forms of online and digital practice including: spreading rumours, ridiculing and/or demeaning another person, casting aspirations on the grounds of race, disability, gender, religion or sexual orientation; seeking revenge or deliberately embarrassing a person by posting intimate photos or videos about them without their consent; accessing another’s social networking profiles with malicious intent and socially excluding a person from a social network or gaming site. This article demonstrates that harm from cyberbullying is a cause for concern for students at each developmental stage and that there are continuities in its appearance that need to be challenged at each point in the educational lifespan. And inaccurately, by university, the idea that ‘nothing can be done’ still is one of the main concerns for the victims. The article concludes with five key recommendations for future research and practice across the educational lifespan.

## 1. The Nature of Cyberbullying 

In the past decade, cyberbullying has emerged as a phenomenon at the school, college and university levels. Definitions of cyberbullying fall into two main categories. Some researchers view cyberbullying as a new form of traditional bullying, following the classical definition originally proposed by Olweus, which states that it is repeated over time, involves a power imbalance between perpetrator and target and has an intention to harm. In fact, Olweus, describes cyberbullying as “an overrated phenomenon”, preferring to view it as simply an extension of traditional bullying into the virtual world. He argues that cyberbullying has low prevalence and that most of those who are being cyberbullied are also being bullied in traditional ways in the ‘real’ world [1]. 

By contrast, other researchers [2,3,4] consider that cyberbullying differs from traditional bullying in distinctive ways since it can invade all aspects of a target’s privacy day and night, both at home and at the educational institution where the target studies. Furthermore, the perpetrators can choose to disguise their identity, so heightening the target students’ insecurity about the quality of their relationships since they may not know which members of their peer group are involved in the cyberbullying. Additionally, if a harmful message “goes viral” through the actions of bystanders who forward the messages to others in their networks, the cybervictim’s distress is compounded. Finally, if, in a ‘one-off’ attack, the action is severe, such as sharing a sexually explicit image of the victim, it transcends the boundaries of cyberbullying and in some countries becomes a criminal offence [5].

From this second perspective, like traditional face-to-face bullying, cyberbullying involves the deliberate intent to hurt a person or persons repeatedly over time. However, researchers such as Schulze-Krumbholz et al. found structural differences in cyberbullying when compared to traditional bullying with an absence of the “pure” cybervictim category in their sample of 6260 adolescents. In their study, they found that the perpetrators of cyberbullying reported that they had been bullied themselves in traditional ways. The researchers speculate that the anonymity of cyberbullying enabled these young people to fight back in ways that would be impossible face-to-face. This study confirms that there is some overlap between traditional and cyberbullying but that the latter has its own distinctive nature [6]. Boulton et al. conclude from their research into bullying at university that findings from traditional bullying should not be generalised to cyberbullying and vice versa. Furthermore, they argue, each broad category (whether bullying or cyberbullying) has sub-types of behaviour, each of which may be distinctive in its own right. They recommend a focus on attitudes towards the behaviour of both bullies and victims to predict whether a student will engage in bullying or cyberbullying in the future [2].

For the purpose of this article, we acknowledge the distinctive features of cyberbullying while at the same time accepting that to some extent there is an overlap between traditional and cyberbullying. In order to move the agenda along and look at continuities across the educational lifespan, and the ever-evolving nature of social media platforms, technology and the internet, we also argue that cyberbullying, as a stand-alone category, has to be considered in order to understand more deeply its potential for social and emotional harm to those directly involved as well as to the witnesses and bystanders. The objective of this paper is to address the following two research questions:

(1) To what extent has cyberbullying *across the lifespan of education*, that is from primary/elementary school, through college to university, been identified as an issue?
(2) What are the implications for educational establishments and educators to reduce and prevent cyberbullying across this learning lifespan?

## 2. Research Findings on Cyberbullying

Most of the research on cyberbullying has taken place in schools and we can gain a great deal of knowledge from this literature about the phenomenon [7]. More recently, there has been an upsurge of interest in cyberbullying among post-16 students, whether in further education (college) or higher education (university), for example, in Canada [8,9,10], in Finland [11,12], in the US [13] and in the UK [14]. The systematic review of cyberbullying among adults by Jenaro, Flores and Frias (2018) provides useful evidence on prevalence, contributing factors, short- and long-term impact and the role of bystanders. Although this review focuses on adults, in practice the authors found that most of the studies had been carried out in college student populations. They conclude that the impact of cyberbullying may be as severe amongst adult populations as amongst children but they note the buffering effect of personal and environmental factors, with emotional intelligence (in the individual) and social support (in the school and community environment) being the most influential variables [15].

Essentially, research into cyberbullying indicates negative immediate effects on the target with potentially harmful long-term impacts on psychosocial development, self-esteem, academic achievement and mental health [16,17,18,19]. For the students who are the targets of such bullying behaviours, the experience is unpleasant and distressing in the short term. However, for some there are longer term negative consequences for their mental health and their academic career. Bullying affects the target’s self-esteem and often leads to social withdrawal from peer-group networks. Consequently, victims of cyberbullying run a heightened risk of mental health disorders, including depression and social anxiety [12,13]. For example, Schenk and Fremouw found that college student victims of cyberbullying were more likely than non-bullied peers to suffer from depression, anxiety and a range of psychosomatic complaints, as well as academic difficulties [20]. Research into the experiences of lesbian, gay, bi-sexual and transgendered (LGBT) students confirms the negative effect of bullying on the mental health of targets. One National Union of Students (NUS) survey [21] found that one in five lesbian, gay and bi-sexual (LGB) students and one in three transgendered (T) students reported at least one form of bullying on campus; many reported that they had to pass as ‘straight’ in order to protect themselves from homophobia and transphobia. Rivers and Valentine et al. report on the extremely negative effect that such treatment had on the mental health of staff and students [22,23]. In extremes, this could lead to suicide.

Cyberbullying involves many, if not all, of the children in a school class since not only the perpetrators and targets are affected but also the witnesses who view negative online messages and harmful video clips and who may in turn forward them to other people for their own amusement, regardless of the feelings of the victim. Cyberbullies are often rewarded for their online behaviour by the approval of the peer group. On the surface, they are the dominant ones with a côterie of admiring followers, but research indicates that the long-term outcomes for them are not good in terms of mental health, social competence and anti-social behaviour [24]. There is a high risk that they will continue to repeat their cyberbullying behaviour [25] since their popularity (which they value) is based on fear and intimidation rather than genuine friendship, making it highly likely that they will continue this method of relating to others as a means of acceptance in their peer group across the educational lifespan.

Cyberbullying among post-16 students continues to cause harmful effects on fellow students. It takes many forms, including such behaviours as: spreading nasty rumours on the grounds of race, disability, gender, religion and sexual orientation; ridiculing or demeaning a person; social exclusion; unwelcome sexual advances; stalking; threatening someone online; revealing personal information about a person that was shared in confidence [14]. West in two studies of cyberbullying among college students in the age-group 16–19 years, found that victims reported such disturbing behaviour as: being told to kill themselves; being sexually harassed; being taunted on account of their religion; being bullied on account of their sexual orientation; being attacked by a ‘gang’ of former friends on Twitter; having nasty comments posted online by a former romantic partner. The emotions experienced by the targets of bullying included anger, hurt, sadness, depression, embarrassment, anxiety, difficulty in concentrating, isolation, self-blame, fear, suicidal thoughts; victims also reported that the cyberbullying had an adverse effect on their capacity to study and on their ability to form social relationships online and in the real world. In the same study, the students who admitted to being cyberbullies reported reasons that included: fun; revenge; anger; jealousy; provocation; desire for power and status; freedom to behave in this way through the anonymity of the social media [26,27]. These findings are confirmed in a larger Canadian study (*N* = 1925) by Faucher et al. (2014) who also noted that women students were more vulnerable to attack through such forms of cyberbullying as “sexting”, “morphing”, “virtual rape” and “revenge porn” [9]. Phipps and Young made similar discoveries in their study of bullying and harassment among UK university students [28].

As indicated in many studies globally and across the educational lifespan, the effects of cyberbullying on mental health can be long-lasting [12,13,29,30]. The public nature of cyberbullying means that it can create a social context where cyberbullying is perceived as “normal” and “acceptable” ([5] p. 1172). Sexting (the exchange of online sexual images or videos) whether generated by others or self-generated, has contributed to recent increases in cyberbullying. This kind of material may begin within a close friendship group or couple but can quickly go viral and be circulated, without consent, to a much larger audience.

## 3. Cyberbullying across the Educational Lifespan

There is, a small, but growing, body of evidence to suggest that there is some continuity in being a bully or a victim from childhood through adolescence and into young adulthood [9,31,32]. Curwen et al. in a retrospective study of 186 university students who admitted to bullying their peers, found that, although there was an overall decrease in the incidence of verbal and physical bullying between high school and university, a substantial proportion of the student bullies in their study had also engaged in bullying behaviour at elementary and high school levels [33]. Chapell et al. found that over half of the adult bullies in their sample had also bullied others during childhood and adolescence. They conclude that this history of bullying indicates long-term benefits so that the behaviour becomes entrenched and continues to be a successful strategy for improving the bully’s social status [34]. Not only that, there is evidence that bullies are popular amongst their peers and that bystanders are often indifferent to the suffering of the victims [25] and that this insensitivity to others’ distress increases over time [35]. The impact on the victims is well documented within the school setting and is emerging as a core focus for looking at cyberbullying within the university sector [31]. The evidence indicates that, while cyberbullying behaviours follow the traditional bullying notions of an imbalance of power, with the added dimension of the internet, the anonymity of the behaviours gives ‘more power’ to the perpetrators and a greater feeling of helplessness to the victims. Additionally, the anonymity of the internet gives perceived power to former victims who can then take revenge on those who tormented them without the repercussions which would happen in a face-to-face context.

Furthermore, we also need to take account of the social contexts where the cyberbullying takes place. As Cassidy et al., argue: “Power imbalances are prevalent in the hierarchical context of universities: administrator to faculty member, senior to junior faculty, tenured to untenured, faculty to staff, supervisor to graduate student, instructor to student, not to mention imbalances based on gender, age, ethnicity, race, social status, and sexual orientation, which can permeate all of those relationships.”([36], p. 2) Indeed, in their research into cyberbullying among students, faculty staff and administrators at four Canadian universities, they link their analysis of cyberbullying to the power and control model, adapted from the field of intimate partner violence, which considers behaviours such as “intimidation, threats, harmful language, social standing, exclusion and harassment in the exercise of power and control over another in a relationship.” ([36], p. 2) Furthermore, they demonstrate that the internet and relevant communication technologies can be used to carry out any, or all, of these forms of behaviour. They conclude that “currently too many students and faculty are suffering the impacts of cyberbullying in isolation, frustrated with the attempts for solving their situations, without any clear guidelines to follow, and within the context of a university culture which benignly seems to tolerate such actions.” ([36], p. 16).

Because of the global reach of the internet, social media platforms and any aggressive online behaviour such as cyberbullying, it follows that research within the university sector is global in its production. Increasingly, researchers in universities reach the same conclusion, namely that cyberbullying is a vast problem and that something has to be done about it [37,38].

## 4. The Role of the Bystander/Witness

Bullying is rarely something that happens only between two individuals, the bully and the victim. Rather, it happens in a social context where bystanders are fully aware of what is happening and take on distinctive participant roles, such as bully, victim, assistant, reinforcer, outsider and defender [39]. In fact, Salmivalli argues that bystanders/outsiders are trapped in a moral dilemma. Although they understand that bullying is wrong, they are acutely aware of their own need for security within the peer group [39]. Many bullies enjoy tormenting a vulnerable peer “for fun” and this can involve entertainment value for the onlookers [40]. The ways in which bystanders respond has a powerful impact on whether the cyberbullying continues or, even worse, goes viral. Through the actions of bystanders, a message that was intended for one other person or for a small group of close friends can quickly be sent to strangers without the permission of the target person. Too often, it is easier for the bystanders to enjoy the spectacle of another’s distress than to challenge the bullies. Bystanders may even blame the victims for not being capable of defending themselves [41]. This indifference, or moral disengagement, is often justified on the grounds that the victim in some way deserved the treatment being meted out to them. In fact, by forwarding distressing images or videos, bystanders collude with the bullies and demonstrate that a process of moral disengagement has taken place [42]. The bystanders’ lack of action to support the victim of cyberbullying can be explained in several ways. They may be afraid of becoming a target in turn if they challenge the bullies or defend the victim and they may justify their actions by rationalising them in terms of blaming the victim [35]. They may even admire the bullies and feel pride in the bullies’ actions [43]. In turn, the actions of bystanders influence the behaviour of the wider peer group, for example, in whether to condone the cyberbullying or to defend the victim publicly. 

However, many bystanders report feelings of discomfort and concern when they witness the occurrence of cyberbullying. Condeza et al. ([44], p. 44) report the following comments from witnesses of cyberbullying at a university in Chile:

“I felt powerless about the offenders’ anonymity, and I felt ignorant because I did not know what to do.”

(female, second year)

“It angered me, but since it was not my friend, I cannot intervene.”

(male, second year)

Additionally, these researchers found that 44.5% of their sample of witnesses reported that they were afraid of becoming cybervictims if they intervened [44].

The problem has become so serious in the UK that Universities UK (UUK) has issued a report, *Changing the Culture*, on sexual violence, harassment and hate crime on campus with a list of recommendations, emphasising prevention, that all universities should take on board, which we will discuss below in the section on the complexities of cyberbullying and the law [45].

Victims’ reactions to cyberbullying are crucial in understanding its incidence and impact. Some researchers argue that there are distinctive roles that can be assigned. For example, Cunningham et al surveyed 1004 university students and found that more than 60% of respondents reported involvement in cyberbullying in the following ways: 45.7% had been witnesses, 5.7% had been victims, 4.9% were perpetrator victims and 4.5% were perpetrators [46]. These are similar roles to the ones suggested by Salmivalli in her research on school bullying [39].

## 5. Victim Coping Strategies in Cyberbullying

Recent research considers cyberbullying in the university setting and its possible relationship with other personal and family variables. For example, Martinez-Monteagudo et al. consider the predictive capacity of the family environment and emotional intelligence in relation to cyberbullying among university students [47]. They sampled 1282 students and found that a deteriorated family environment increased the probability of being both a victim and a perpetrator of cyberbullying. Furthermore, they also found that the emotional intelligence of the students had a correlation with predicting the levels of participation in cyberbullying. They argue that: “…part of the problem of cyberbullying in the university setting may depend on the quality of the student’s family relationships and EI level. These are variables that have yet to receive much attention from the scientific field. Thus, the family is seen to play a relevant role as a protective factor from cyberbullying, even in a university setting, controlling the behaviour of its members and the use of new technologies. Similarly, EI acts as a protective factor to prevent students from being victims as well as aggressors, highlighting the fundamental role exercised by emotional regulation in students. Thus, it is corroborated that both variables are relevant factors to be taken into consideration when developing social and educational policies, and when developing intervention programs to alleviate this problem.” ([47] p. 224).

Such research demonstrates the continuities and patterns across the educational lifespan, but crucially also suggests that even though the students are at university, there is still an element of family involvement and engagement that will help the student if they become involved in a cyberbullying scenario. This is also pertinent since an increasing number of students choose to live at home whilst they study to keep down the costs and expenditure of being at university. Assumptions that students are over 18 and able to defend for themselves need to be challenged as positive relationships with the family emerge as a coping mechanism for involvement in cyberbullying, as they do for school students.

Attachments to members of the peer group are also significant, as McCloughlin et al. found in their survey of Australian adolescents [48]. Young people in this age-group have a strong need for social connectedness. Those who were more socially connected had better mental health than those who were not and had more effective defences against cyberbullying when it happened. The less young people feel socially connected to their peer group, the lower their self-esteem and the greater their sense of loneliness, both offline and online [49].

Gender also plays a very significant role in cyberbullying across the educational lifespan, notably at the university level. Cunningham et al., found that male students were more likely than their female counterparts to report as perpetrators or bully-victims [46], while Boulton et al., argued that the male undergraduates in their study viewed cyberbullying and those who perpetrate it, less negatively than the female students did [2].

Younger victims may try to fight back (often unsuccessfully) against the bullies or express their distress through tears and emotional displays, so risking further physical and psychological attacks from the bullies, and contempt from bystanders [50]. As victims progress through the educational lifespan, they are less likely to retaliate actively but are more likely to employ passive tactics, such as blocking or ignoring. As Kernaghan and Elwood argue in their qualitative study of cyberbullying during early adolescence, the cyberbullying event becomes a performance where the bystanders take the role of audience to a drama. The more the victims become distressed, the greater the entertainment and the less likely that bystanders will intervene to help [51]. Similarly, in focus groups, the adolescents in the study by Purdy and York reported that during an episode of cyberbullying, it is often easier to go along with the crowd since they were afraid that the bullies would turn on them [35]. Understandably, victims learn to disguise their true feelings. As children become adolescents, they are less likely to engage in fighting fire with fire, perhaps because of such negative experiences from peers and perceived inaction from adults. By the time cybervictims are at university, while they may have learned that aggressive retaliation achieves little, many have failed to find alternative coping strategies to reduce or prevent the cyberbullying [52].

A small number of studies indicate that the university sector could be better equipped to deal with cyberbullying, with lecturers and other staff becoming more proactive in helping. When considering victims of cyberbullying and how they deal with the situation whilst at university research is scant, in comparison to the school setting. Nonetheless there are some illuminating studies. For example, Orel et al., in a sample of 282 students, investigated the coping strategies the students would potentially use in response to future cyberbullying incidents. Blocking of the perpetrator was found to be the most common tactic considered [53], but does this passive tactic solve the problem?

Research from the school setting provides strong evidence that telling someone about being bullied is a crucial first step to resolving the problem, however, studies of college and university cyberbullying find that victims feel, at university, that telling someone about their situation will not result in a satisfactory outcome. For instance, Alqahtani et al., in their study of 165 university students found that 49% of students felt that their university could not or would not do anything about cyberbullying, even if it was reported; 47% felt that university staff would not believe or understand them even if they did complain or report what had happened [54]. Thus interventions that actively encourage children to tell someone that they are being cyberbullied, for example a member of staff from the pastoral care team, a school nurse or a peer supporter, are not being carried over to FE and HE levels so therefore are not being practiced across the educational lifespan. 

## 6. The Victim-Offender Cycle

In Finland, Pörhölä [25,55] found that nearly half of those who reported being bullied at university had previously been subjected to school bullying. Retrospective studies, for example, Bauman and Newman in the US, also indicate that there is a high likelihood that university students who report being bullied were also bullied at junior and high school levels, but that being a stable victim from junior high to high school and then to university was more characteristic of male than female students [56]. However, the distinctive participant roles that are often researched and reported in more traditional forms of bullying [39] are not always as clear cut in cyberbullying incidents. Some researchers [2] have argued that it is almost impossible to make the distinction between the ‘bully’ and the ‘victim’ in the online world where cybervictims hide behind anonymity to become cyberbullies in revenge. Similarly, at secondary school level, Schultze-Krumbholz, et al., found that adolescents who bully others online are very likely to have been victims themselves [6]. Cyberbullying research confirms some degree of continuity across the educational lifespan but there are also discontinuities that need to be further investigated. 

The discipline of criminology potentially offers useful insights since the ‘victim/offender’ cycle is played out in different criminal scenarios and the distinction between roles is not that clear cut. Decades of research have shown that there exists a connection between the perpetrator and the target, which has a number of complicated dynamics that need further investigation [57], with some arguing that the overlap is developmental in its nature and predictable from childhood [58] and others arguing that the relationship is so “intimately connected” that trying to understand victims and offenders as separate entities is not possible [59]. Clearly, the relationship between cyberbully and victim is key to understanding the wider cultural context and significance of the act [60] and is an area of further study that is needed when considering cyberbullying across the educational lifespan.

The victim/offender relationship is a difficult area to unpack when it comes to cyberbullying and research is beginning to show that it becomes even more difficult within the university sector because of the age and potential previous experience of all those who are involved. One way to begin to understand the complex interactions is to look at reactions to cyberbullying when it occurs. For example, Eristi and Akbulut surveyed 567 undergraduate students and 211 high school students. Among this sample they found that 170 (29.98%) of the undergraduates and 120 (56.87%) of the school aged students had been cyberbullied within the last six months prior to the study. They then considered and investigated the behavioural cyberbullying reactions of victimised students under four key factors, which were revenge, countermeasure, negotiation and avoidance. They also considered victimized students’ emotional reactions as either internalizing (for example, ‘fear’, ‘panic’, ‘anxiety’ ‘embarrassment’ or ‘guilt’) or externalizing (for example, ‘seeking revenge’, ‘getting aggressive’ or ‘angry’). Their research demonstrated that behavioural and emotional reactions varied according to gender and whether they were in school or at university [61]. Furthermore, computer self-efficacy and internet use were associated with different reaction types. They conclude that: “...new model and intervention proposals directed at reducing the unpleasant/damaging victimization consequences may better serve the goal of mitigating these consequences than descriptive investigations.”([61], p. 9) Although this is a relatively large scale survey in an emerging field of investigation, it highlights, and adds to the debate, that victim reactions and retaliation processes must be included when considering how to understand cyberbullying across the educational lifespan. 

## 7. Current Policy and the Role of the Law—The UK Context

The issues around law, the boundaries of responsibility and criminalization of behaviours are to be considered country by country, due to the nature of individual criminal justice systems. Here, we look at the UK as an example to highlight the minefield of problems that are emerging around cyberbullying and its intersection with crime. Although the authors acknowledge that there are many different laws and practices within other jurisdictions, focusing on one system will highlight the complexities of not tackling cyberbullying across the educational lifespan.

Within the UK, Advance HE, who were appointed as independent evaluators for the Office for Students (OfS) Catalyst-funded projects, were tasked with looking at ‘what works’ in student engagement in safeguarding projects following the UUK Changing the Culture Report [45] follow up report *Changing the Culture: One Year On* [62] and subsequent Catalyst funding to evaluate good practice across the sector. So far, they have identified the following as being the most effective ways to achieve this: firstly, “co-creation with students of training content and campaigns”, secondly, “Peer-to-peer learning or mentoring” and thirdly, “Collaboration with Student Unions.” ([63], p. 2). They strongly recommend that students themselves should be encouraged to re-engage with practices, such as peer support and peer mentoring, that are commonplace in most primary and secondary schools in some form or another [64]. Perhaps rather than re-invent the wheel, the further and higher education systems need to reintroduce practices and policies to their students that the majority would have had some knowledge about whilst in their earlier years of their education.

Furthermore, UUK has now uncovered many issues that should be taken into consideration when focussing on sexual misconduct, hate crime and harassment, the original remit of *Changing the Culture* [45]. In October 2018 they held a round table event to explore the nature and scope of online harassment and cyberbullying within the university sector and to see what more can be done to prevent and respond to this form of hate crime. A report will follow in April 2019. Therefore, cyberbullying within universities is under consideration but it is still key to remember that any recommendations that are made are not mandatory as there is not a regulatory body that enforces universities to develop centralised policies. This is in contrast to schools and FE colleges that are required by law to have safeguarding practices in place and anti-bullying policies. 

The problem here is the age of the students and, as has been argued in previous research, once students are at university, they are over the age of 18 and are legally adults, which brings with it challenges of responsibility and boundaries of policing [5]. It is worth noting here that although cyberbullying is not a criminal offence, there are a number of laws and relevant legislation that are used by the Crown Prosecution Service (CPS) to prosecute cases that involve online communication in England and Wales. These include: Offences Against the Person Act (1861), Protection from Harassment Act (1997), Malicious Communications Act (1988), The Crime and Disorder Act (1998) and the Serious Crime Act (2015) Students over the age of 18 can potentially be prosecuted for cyberbullying related offences. Since many students are unaware of this legislation, we recommend that education on the legal consequences of actions must be bought into the curriculum at a much earlier level. If behaviour is learned and repeated across the educational lifespan, the legal aspects need to be dealt with from the beginning, as soon as children and young people are able to understand the implications of what they are doing [49]. It would appear to be the case that certain communities within the wider society have a very high tolerance level for what is considered ‘normal’ or ‘acceptable’ in terms of violent behaviours. For example, within the UK amongst teenagers, sexting, upskirting and revenge porn have become particular causes for concern [5,65,66] and this is especially pertinent in the online world. The boundary between ‘flirty’ behaviour and crime is becoming even more blurred. Therefore, this needs to be factored in along with challenging a lack of knowledge about the law. It needs to be embedded in education, across the educational lifespan, with legal awareness training.

## 8. Intervening to Prevent and Reduce Cyberbullying

International evaluation studies [67,68] have systematically reviewed the successes and failures of interventions to combat bullying/cyberbullying at school level. For example, Ttofi and Farrington, in their meta-analysis of 44 school-based interventions carried out over a period of 25 years in Europe, Australia, America and South Africa, found that certain intervention programmes reduced rates of bullying others by 20–23% and victimisation by 17–20% [68]. The key components of such programmes included parent meetings and training, consistent disciplinary methods, classroom rules, school conferences and skilled classroom management by teachers. Smith et al. (2016) examined the effect of disciplinary methods and concluded that these needed to be non-punitive, negotiated by adults and children, and restorative in nature, with an emphasis on school safety for all, on the promotion of a positive school ethos, and on positive relationships throughout the school. [67]. The evidence pointed to the effectiveness of sanctions that were perceived by the students as fair and reasonable, arising from a process of consultation in which members of the school community had taken part. Purdy and York, focussing on cyberbullying, argue that new ground rules are emerging concerning responsibility for action to counteract cyberbullying since the bullying frequently occurs at home during evenings and weekends when it is parents who have responsibility for their child’s behaviour [35].

Within the school setting, bullying and cyberbullying interventions typically engage in a ‘whole school’ approach considering other factors such as the family and wider community in addressing the problem. Many schools across the world now adopt Social and Emotional Learning (SEL) programmes to enhance children’s and young people’s empathy for the distress of others and to create a more pro-social climate in the classroom and the school at large (for a comprehensive international overview, see [69]). An international review of successful and unsuccessful interventions [70] identified key components in the reduction of bullying, to include the creation of a whole-school policy against bullying and meetings/workshops with parents. Smith et al. (2016) identified the effectiveness of restorative methods within the school and the community in order to create a co-operative school climate with an emphasis on positive relationships and active participation on the part of students and staff in the creation and implementation of anti-bullying action [67]. Going one step further, Finne et al. argue that the dynamics of bullying are likely to persist in a school context if only superficial anti-bullying action is taken [71]. They argue that some form of rehabilitation needs to take place to change the quality of “peer ecology” ([71] p. 356) in the classroom. They argue that the moral disengagement that occurs when bullying persists creates a group norm that may well continue even when the active forms of bullying have ceased as a result of a successful intervention. Their model of relational rehabilitation takes three steps:

(1) *Ensuring teacher authority*: teachers play a crucial role through an authoritative teaching style that emphasises warmth, acceptance and tolerance. This can counteract the destructive power of the bullies;
(2) *Redistribution of social power and promoting a supportive classroom community*: In an abusive class community, power will have been wielded by the bullies. The teacher plays a crucial role in breaking up the previous hierarchies and creating a fairer social system that promotes friendship and prosocial behaviour. Such a class culture will be tolerant of difference and open to discussions about social inclusion and caring. The teacher plays an active part in helping the pupils to repair the damage that has been done.
(3) *Providing social and emotional learning (SEL) to the whole class*: Whole-class SEL programmes take a variety of forms but their essential principles concern the inclusion of all, the valuing of prosocial behaviour and the creation of a warm, supportive classroom community.

Similarly, in the college and university sector, the idea of rehabilitation is beginning to be discussed, with a growing emphasis on changing the culture to one of tolerance and community [62,72]. However, there is not to date the same level of coherence nor shared practice as appears at school level.

Whilst a number of studies focus on reporting the levels of cyberbullying across the educational lifespan, there are huge variations in how the problem should actually be tackled. Following the recommendations from cyberbullying within the school setting, research within the university sector is beginning to acknowledge that a very important and often neglected way to understand and deal with the problem is to engage with the students themselves. Cunningham et al (2015) stipulated that there were five key areas that need to be considered when tackling cyberbullying within the university sector:

(1) Emphasise the impact of cyberbullying on victims;
(2) Change cyberbullying prevention attitudes;
(3) Teach anti-cyberbullying strategies;
(4) Enable anonymous online reporting;
(5) Combine prevention with consequences ([46], p. 380).

These recommendations were made by the students themselves and the authors conclude that they would help when designing and implementing university anti-cyberbullying programmes. Such research and subsequent recommendations indicate the need for a rather more complex whole university sector approach to tackling the problem than is currently available. Furthermore, the involvement of the students themselves is crucial, especially given the age of undergraduate students. They are adults and they need to be consulted in what works to reduce cyberbullying and thus make their learning environment more positive, especially in the light of the move to a heavy reliance on the online world for everything within the university sector, from online registration and timetables, to attendance monitoring, assignment submission and virtual learning environments. Universities continue to advance in adapting online systems and practices. Thus, it follows that mechanisms could also be introduced and improved to support students who have to use and engage with the online world.

## 9. Conclusions

*To what extent has cyberbullying across the lifespan of education, that is from primary/elementary school, through college to university, been identified as an issue?*

The evidence from the literature that we reviewed indicates that cyberbullying across the educational lifespan continues to be a critical issue for a proportion of students. Despite the wealth of high-quality research over three decades, the problem still needs to be addressed as a matter of urgency. Cyberbullying, to some extent, is a continuity and attitudes towards it need to change. While there are some overlaps with traditional bullying, there are clearly aspects unique to the online existence that have to be considered. Traditional anti-bullying interventions may not always be sufficient to capture the complexities of cyberbullying. The following gaps in knowledge need to be considered:

(1) One important gap concerns the widespread lack of knowledge amongst educators and their students about the legal consequences of cyberbullying behaviour across the educational lifespan.
(2) A second concerns the finding that cyberbullying is distressing at all stages of the educational lifespan but there is a lot of evidence now to support the view that cybervictims become less likely to report it as they reach FE and HE levels. The gap in understanding appears to be at university level and the assumption that students as young adults no longer need support in dealing with cyberbullying.
(3) Furthermore, the bystanders express less empathy and more moral disengagement the older they get, with university students showing the least sensitivity to peers’ distress.
(4) Finally, by the time they reach adolescence and young adulthood cybervictims have run out of options for the help and support which they are more likely to get at primary school but which are less likely to feature as they progress through the educational lifespan They can resort to acceptance of their fate with all the attendant mental health difficulties that this entails *or* they can take revenge on their tormentors by becoming cyberbullies themselves. Neither is a good coping strategy. The gap here is we need more information on successful and unsuccessful strategies adopted by cybervictims across the educational lifespan.

*What are the implications for educational establishments and educators to reduce and prevent cyberbullying across the educational lifespan?*

Looking at cyberbullying in educational silos is not helpful. Students do not arrive at university and their problems begin. Rather, looking at education as a longitudinal journey is more helpful. Good practice could and should be shared amongst schools, colleges and universities in order to safeguard and educate students at all levels of their education. The following action points emerged from the literature search:

(1) At primary school level and to an extent at secondary level, schools are increasingly developing Social Emotional Learning (SEL) [69], restorative practices [73] or emotional rehabilitation [71] interventions to address the issue of cyberbullying, with an emphasis on the destructive nature of moral disengagement. Peer support systems are widespread [64]. Participant role theory is also successfully used in Finland [39]. However, at FE and HE levels this is not happening. We have already mentioned the work that UUK [62] advocated around bystander training but to date this is only used in a few institutions and is yet to roll out across the sector. For those students that do identify as victims, there need to be improved systems, such as student welfare and counselling, to help them. Perhaps what need to be introduced at the FE and HE level are more comprehensive pastoral systems with appropriate training for the staff involved.
(2) While there is a strong emphasis at school level on social skills, the development of empathy and the need for restorative practice to mediate in disputes, the legal aspects of cyberbullying are scarcely mentioned. By contrast, legal aspects of cyberbullying are increasingly on the agenda at FE and HE levels since the students are by now young adults. When cyberbullying incidents become criminal acts, the police are involved and legal proceedings may ensue. This indicates an imbalance at different levels of the educational lifespan. What is needed is legal awareness training across the educational lifespan.
(3) There is strong evidence of both continuities and discontinuities in cyberbullying across the educational lifespan but, to date, there is little information on the nature of successful and unsuccessful coping strategies used by the targets of cyberbullying. Consequently, researchers and practitioners need to continue to collaborate to design methods for equipping future generations with the tools to navigate the internet safely and, in a socially positive way.
(4) Primary and secondary schools have invested a great deal in counteracting the harmful effects of xenophobia, misogyny and homophobia; this is less evident at FE and HE levels. There should be considerably more sharing of expertise between schools, colleges and universities about interventions that are effective in this domain.
(5) Finally, the literature indicates an urgent need to consider cyberbullying in its cultural context–its social and environmental ecology. The issue of moral disengagement is scarcely mentioned in the curriculum or in the pastoral care systems at HE and FE levels despite the evidence of cultural issues such as “laddism” [28], xenophobia [74], sexism [10,49] and homophobia [22] in FE and HE settings. Cyberbullying needs to be addressed and understood in all of its cultureal contexts across the educational lifespan.

One thing that is for certain is that the internet is not going to disappear. Technology will evolve, and more social media platforms will emerge. What this article shows is the urgent need to address certain gaps in the literature and implement successful interventions right across the educational lifespan to tackle cyberbullying.
